# Supplementary material for: Hippocampal transcriptome profiling reveals common disease pathways in chronic hypoperfusion and aging
Source: Aging (Albany NY). 2021 Jun 1;13(11):14651–74. doi: 10.18632/aging.203123 (PMC8221317; doi:10.18632/aging.203123)
Supplement: Supplementary Tables 4 and 5 [file aging-13-203123-s006.pdf]

SUPPLEMENTARY TABLES

Supplementary Table 4. List of all KEGG pathways under the pathway enrichment analysis for mice subjected to 7 and 30 days of BCAS.

Description	ID	q-value	geneID	Count
MAPK signaling pathway	mmu04010	0.002396	Gna12/Cacna1e/Hspa1l/Gadd45b/Fos/Gadd45g/Rasgrf2/Cacna2d3/Nr4a1/Cacna1h/Dusp1/Pdgfrb/Fgfr2/Ikbkb/Pla2g3/Ntf3/Prkca/Prkcb/Fgfr3/Cacnb2/Mapk3	21
Gastric acid secretion	mmu04971	0.00249	Car2/Itpr2/Gnai2/Plcb2/Kcnj2/Chrm3/Sstr2/Prkca/Ezr/Prkcb	10
Long-term depression	mmu04730	0.007753	Gna12/Itpr2/Ryr1/Gnai2/Pla2g3/Plcb2/Prkca/Prkcb/Mapk3	9
Regulation of actin cytoskeleton	mmu04810	0.011272	Gna12/Itgb4/Iqgap2/Pdgfrb/Fn1/Gsn/Nckap1/Fgfr2/Itga11/Itga9/Chrm4/Chrm3/Ezr/Fgfr3/Mapk3/Chrm5	16
Calcium signaling pathway	mmu04020	0.013768	Cacna1e/Plcg1/Cacna1h/Pdgfrb/Phkg1/Itpr2/Ryr1/Phka1/Drd5/Plcb2/Chrm3/Prkca/Prkcb/Chrm5	14
Axon guidance	mmu04360	0.026275	Efnb3/Sema6a/Slit1/Epha7/Sema3c/Plxna4/Srgap3/Gnai2/Epha6/Mapk3/Sema3e	11

Supplementary Table 5. List of all significantly (q<0.05) enriched upregulated and downregulated GO terms for mice subjected to 7 and 30 days of BCAS.

Description	ID	Category	q-value
cellular response to growth factor stimulus	GO:0071363	BP	0.007679
positive regulation of endothelial cell migration	GO:0010595	BP	0.007679
regulation of endothelial cell migration	GO:0010594	BP	0.009149
endothelial cell migration	GO:0043542	BP	0.009149
positive regulation of neuron death	GO:1901216	BP	0.009149
posttranscriptional regulation of gene expression	GO:0010608	BP	0.009149
regulation of cell morphogenesis	GO:0022604	BP	0.009149
protein serine/threonine kinase activity	GO:0004674	MF	0.015801
negative regulation of protein phosphorylation	GO:0001933	BP	0.023866
modulation of synaptic transmission	GO:0050804	BP	0.023866
postsynapse	GO:0098794	CC	0.026809
negative regulation of protein modification process	GO:0031400	BP	0.029445
regulation of cellular amide metabolic process	GO:0034248	BP	0.029445
regulation of mitochondrial fission	GO:0090140	BP	0.033902
negative regulation of phosphorylation	GO:0042326	BP	0.048738
